# Psychopathological Variables and Sleep Quality in Psoriatic Patients

**DOI:** 10.3390/ijms17071184

**Published:** 2016-07-21

**Authors:** Maria Luca, Antonina Luca, Maria Letizia Musumeci, Federica Fiorentini, Giuseppe Micali, Carmela Calandra

**Affiliations:** 1 Psychiatry Unit, Department of Medical and Surgical Sciences and Advanced Technologies, University Hospital Policlinico-Vittorio Emanuele, Santa Sofia Street 78, Catania, 95100 Sicily, Italy; lucmaria@tiscali.it; 2 Department of Medical and Surgical Sciences and Advanced Technologies, University Hospital Policlinico-Vittorio Emanuele, Santa Sofia Street 78, Catania, 95100 Sicily, Italy; antolucaster@gmail.com; 3 Dermatology Clinic, University of Catania, University Hospital Policlinico-Vittorio Emanuele, Santa Sofia Street 78, Catania, 95100 Sicily, Italy; marialetizia.musumeci@virgilio.it (M.L.M.); federica.fiorentini88@gmail.com (F.F.); gimicali1@hotmail.it (G.M.)

**Keywords:** psoriasis, depression, anxiety, sleep quality

## Abstract

Psoriasis is an inflammatory disease frequently associated with psychiatric disturbances and sleep disorders. The aim of the study was to assess the prevalence of depression, interaction anxiety, audience anxiety, and sleep quality in psoriatic patients. One hundred and two psoriatic patients were enrolled and underwent the following questionnaires: Zung Self-Rating Depression Scale (SDS), Interaction Anxiousness Scale (IAS), Audience Anxiousness Scale (AAS), Pittsburgh Sleep Quality Index (PSQI). The severity of skin lesions was assessed by Psoriasis Area Severity Index (PASI). The presence of a link between clinical variables and with demographic data has been investigated. Psoriasis was linked to depression, interaction and audience anxiety, as well as to poor sleep quality; 37.5% of patients were depressed, 46.1% scored above 37 at the IAS, 47.1% scored above 33 at the AAS. Thirty-nine subjects (38.2%) presented a PSQI ≥ 5. An association between interaction anxiety and lower limbs psoriasis-related erythema as well as between PSQI and head psoriasis-related erythema was found, particularly among male patients. Hence, psoriatic patients should be assessed from a holistic point of view, in order to identify associated disorders that could benefit from targeted treatments.

## 1. Introduction

Psoriasis is a chronic inflammatory skin disease affecting 2%–4% of the general population in Western countries [1,2]. It is clinically characterized by typical raised scaling and red papules and plaques mostly affecting the scalp, elbows, knees, and lumbosacral area (plaque psoriasis). Less-frequent clinical subsets—accounting approximately for 10% of cases—include guttate psoriasis, characterized by small tear shaped scattered papules, pustular psoriasis, and variants affecting atypical regions (flexural/inverse psoriasis, palmoplantar psoriasis, nail psoriasis, mucosal psoriasis). Unstable psoriasis can lead to rare complications (erythrodermic and generalized pustular psoriasis) [2]. The burden of this skin disease is considerably high in terms of social costs [3], patients’ quality of life, and high rate of comorbidity (hyperlipidemia, hypertension, diabetes, arthritis) [4,5,6]. Several factors may play a role in its occurrence and progression, among which are genetic determinants, immunological factors, diet, and voluptuary habits [7]. Particular attention has been given to the existing link between psoriasis and psychosocial stress. In fact, on one hand psychosocial stress represents a risk factor for this invalidating skin disease [8] but, on the other hand, psoriasis itself dramatically affects the patients’ self-image and causes a considerable psychosocial morbidity [9]. Suffering from psoriasis implies feeling stigmatized, dealing with reduced working possibilities, experiencing shame and worry [10,11]. Psoriatic patients present high rates of anxiety and depression and are at risk of suicidal behavior [12,13]. Some authors reported that depression is independently associated with psoriasis, no matter the severity of skin lesions [14]. Moreover, psoriasis significantly affects interpersonal relationships and sexual life [15]. In fact, social avoidance is one of the dreadful consequences of psoriasis and leads to a progressive impairment of the patients’ social functioning [16]. In addition, pruritus and pain have been related to the sleep disturbances often experienced by patients, so that it has been suggested to investigate on the presence of restless leg syndrome and insomnia in every single case [17,18]. Hence, the patients should be assessed from a holistic point of view. In fact, the evaluation of both clinical and psychological variables would allow a careful selection of patients which could take advantage from psychological interventions [19]. Despite this, studies simultaneously evaluating psychopathological alterations in psoriatic patients while paying attention to demographic and clinical variables are relatively scant. The aim of this study was to assess the prevalence of psychiatric disturbances and sleep disorders in psoriatic patients, also taking into account demographic and clinical variables (see below).

## 2. Results

### 2.1. General Characteristics of the Patients

One hundred and two psoriatic subjects were consecutively enrolled in the study. The mean duration of psoriasis was 15.1 ± 12.3 years. Forty-five subjects (44.1%) presented comorbidities. Among them, five (4.9% of the whole sample) suffered from anxiety and were under treatment with benzodiazepines. For more details, see Table 1.

### 2.2. Dermatological Characteristics of the Patients

**Figure 1:**
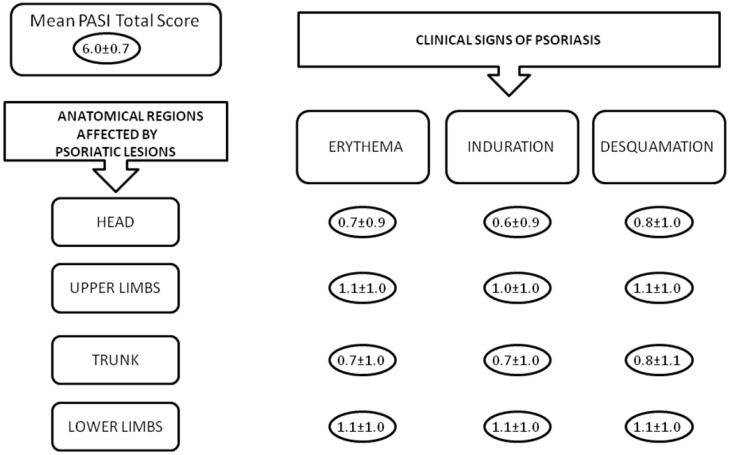
Psoriasis Area Severity Index (PASI) score. The figure shows the mean values of the PASI total score as well as its single items.

The mean Psoriasis Area Severity Index (PASI) total score was 6.0 ± 7.2. For more details on the PASI single items, see Figure 1. Seventy-eight (76.5%) subjects were under remission and presented a PASI = 0, 18 (17.6%) presented a PASI between 0.1 to 20, and 6 (5.9%) showed a PASI > 20. Forty-two (41.2%) subjects presented psoriatic arthritis. Fifteen (14.7%) were under treatment with narrow-band ultraviolet-B light, 40 (39.2%) with biologic therapy, 16 (16.2%) with systemic therapy (cyclosporine, acitretin, or methotrexate), and 26 (25.5%) with topical therapies. All the treated subjects were under therapy for at least 2 months. Five subjects (4.9%) were untreated.

### 2.3. Psychiatric Characteristics of the Patients

The patients presented an average Zung Self-rating Depression Scale (SDS) score of 37.3 ± 9.6. The average standardized *z* score was 46.7 ± 12. Thirty-eight (37.5%) patients were found to be depressed (*z* score > 50). The Interaction Anxiousness Scale (IAS) mean score was 37.3 ± 10.3, with a median of 37. Forty-seven (46.1%) patients scored above the median IAS. The Audience Anxiousness Scale (AAS) mean score was 32.9 ± 10.3, with a median of 33. Forty-eight (47.1%) subjects scored above the median AAS.

### 2.4. Sleep Quality among the Patients

The Pittsburgh Sleep Quality Index (PSQI) mean score was 4.4 ± 3.6 with a median value of 3. Fifty (49%) subjects scored above the median PSQI. Thirty-nine subjects (38.2%) presented a PSQI ≥ 5.

### 2.5. Pruritus and Sleep Quality

It has been investigated whether pruritus was perceived as a cause of poor sleep quality by our patients. Six subjects (5.9%; 4 M, 2 F) specifically reported psoriasis-related pruritus as a condition disturbing their sleep (open answer of the PSQI). Of them, three presented diffuse skin lesions [Head (H), Upper Limbs (UL), Trunk (T), Lower Limbs (LL)], in the other three the lesions were confined to the limbs.

### 2.6. Gender Differences

Forty-nine females (mean age 52.2 ± 13 years) and 53 males (mean age 45.5 ± 13.6 years; *p* = 0.013) were enrolled. No statistically-significant gender differences in terms of education, PASI total score, or IAS were found. Females presented a significantly higher score on the SDS (41 ± 9 vs. 33.8 ± 9; *p* < 0.0001), on the AAS (35.1 ± 10.8 vs. 30.8 ± 9.5, *p* = 0.04) and at the PSQI (5.3 ± 4.1 vs. 3.5 ± 2.8, *p* = 0.009) than males.

### 2.7. Pearson’s Correlations

The PASI total score did not show correlations with duration of psoriasis, Body Mass Index (BMI), psychopathological variables (SDS, IAS, AAS) and sleep quality (PSQI). No correlation has been found neither among psychopathological variables, nor between them and sleep quality or occupation.

### 2.8. Unconditional Logistic Regression

No association between SDS, AAS, and PASI (in terms of both total score and clinical signs of psoriasis: erythema, induration, desquamation) has been found. Analyzing the psychopathological variables (SDS, IAS, AAS) and the PSQI, no association between these variables and disease duration, presence of psoriatic arthritis, comorbidities, age, years of education, occupation, BMI, or therapy was detected. An association between the IAS score and lower limbs psoriasis-related erythema was found (OR 2.8; 95% CI 1.08–7.2; *p* < 0.033). This association was stronger when analyzing males alone (OR 6.0; 95% CI 1.12–32.4; *p* = 0.035). In addition, PSQI score has been found to be associated with head psoriasis-related erythema (OR 14.8; 95% CI 1.4–147.2; *p* < 0.021). This association was stronger when analyzing males (OR 38.2; 95% CI 1.03–1412.2; *p* = 0.04). For more details, see Table 2 and Table 3.

## 3. Discussion

Our findings support a link between psoriasis and psychopathological disturbances. In fact, nearly 40% of patients were depressed, independent of psoriasis severity. As a matter of fact, our sample was largely represented by patients with an optimal disease control (PASI 0). Other studies have demonstrated high rates of depression among psoriatic patients, regardless of psoriasis severity [14]. The association between depression and duration of psoriasis found by some authors [12] was not noticed in our patients. As reported in previous studies [20], female patients were more likely to be depressed than males (SDS 41 ± 9 vs. 33.8 ± 9; *p* < 0.0001). Our results remark that the strong link between psoriasis and psychopathological disorders should not be underestimated, since psoriatic patients experiencing a psychological vulnerability are often “stress reactors” and show higher levels of anxiety and depression [21]. In addition, these patients frequently present outbursts of psoriasis and being in search of social approval are even more distressed by the disease [22]. They also show an impaired disease-related quality of life and are more prone to tobacco and psychotropic medications use [23].

To the best of our knowledge, this is the first study specifically assessing interaction and audience anxiety among psoriatic patients. It is worthy of consideration that the 46.1% of the patients scored above 37 on the IAS and the 47.1% scored above 33 on the AAS. Audience anxiety was more severe among women (AAS 35.1 ± 10.8 vs. 30.8 ± 9.5, *p* = 0.04). It is sadly known that psoriatic individuals often experience feelings of shame, stigmatization, and social fear [16,20,24]. An interesting finding of this study pertains to the association between interaction anxiety and LL psoriasis-related erythema (OR 2.8; 95% CI 1.08–7.2; *p* < 0.033), stronger among male patients (OR 6.0; 95% CI 1.12–32.4; *p* = 0.035). As far as we know, other studies evaluating the link between selected clinical psoriatic features and psychological symptoms are not available. Hence, the explanation of this finding is purely speculative. The higher level of interaction anxiety in patients with LL involvement could be explained by psoriatic localization in the genital area, which is included in the LL PASI region. Moreover, genital psoriasis—which severely impacts sexuality—is not infrequent, particularly among males [25,26]. Unfortunately, prejudices towards psoriasis still exist and many people believe that it is an infective disease related to poor hygiene; hence, they are disinclined to have sexual intercourse with a patient affected by psoriasis [27]. Another interesting finding is represented by the high percentage of poor sleepers among our psoriatic patients, independent of depression severity. In fact, 38.2% of the patients suffered from poor sleep quality (PSQI ≥ 5). Females slept worse than males (PSQI 5.3 ± 4.1 vs. 3.5 ± 2.8, *p* = 0.009). This finding confirms the association between psoriasis and sleep disturbances, mostly re-conducted to pruritus and pain [17]. In our study, the PSQI score showed a significant association with H psoriasis-related erythema (OR 14.8; 95% CI 1.4–147.2; *p* < 0.021) when considering the whole sample, with a stronger association in males (OR 14.8; 95% CI 1.4–147.2; *p* < 0.021). A possible explanation could lie in the occurrence of pruritus. An H involvement was found to be present in half the subjects reporting pruritus at the open answer of the PSQI. Nevertheless, scalp psoriasis is often reported in the literature as an intensely pruritic condition [28]. It is worth considering that the threshold for pruritus is lower during the evening and night. In fact, pruritus follows a circadian rhythm related to several factors including hormones (lower cortisol levels in the evening) and skin characteristics (impaired epidermal barrier function, increased distal-to-proximal skin temperature gradient) [17]. It has been reported that the core circadian gene, Clock, could be involved in psoriasis-like skin inflammation since it regulates the expression of interleukin 23R. In mice, a loss-of-function mutation of Clock was protective against imiquimod-induced dermatitis. On the contrary, the same mutation of Per2—another gene of the circadian system inhibiting Clock—was related to a worse response to imiquimod [29]. Many studies suggested an altered circadian rhythm in psoriasis—in light, for example, of the findings of disrupted rhythms of blood pressure and heart rate among psoriatic patients [29]. However, the link between sleep disorders and psoriasis is still controversial [30]. Some evidence suggests that psoriasis can be directly and indirectly linked to sleep disturbances which, in turn, affect psoriasis. In fact, psoriasis is characterized by a dysregulation of cytokines and thermoregulation that may result in nocturnal pruritus and impaired sleep induction [31]. Many molecular mediators seem to be involved in the pathogenesis of pruritus. A dysfunctional expression and/or distribution of a great number of neuropeptides (e.g., substance P, somatostatin, vasoactive intestinal peptide) has been demonstrated in psoriasis. This condition leads to the activation of the immune system and of the inflammatory cascade through the stimulation of pro-inflammatory cytokines. Among the latter, interleukin 2, as well as prostaglandins, seems to play an important role in the pathogenesis of pruritus. The alteration of the vascular skin system occurring in psoriasis could also be responsible for pruritus [32]. Conversely, sleep deprivation leads to the activation of the immunological-inflammatory cascades, thus determining the worsening of the skin lesions [33]. Moreover, it is known that psoriasis is frequently associated with disorders negatively affecting sleep quality, such as obesity, diabetes, and hypertension [17]. The main limit of this study is the lack of a control group. Therefore, the findings need to be confirmed in larger case–control studies.

## 4. Experimental Section

### 4.1. Materials and Methods

Psoriatic outpatients have been consecutively enrolled in the Dermatology Clinic of our University Hospital. All subjects gave their informed consent for inclusion before they participated in the study. The study was conducted in accordance with the Declaration of Helsinki, and the protocol was submitted to the local Ethics Committee. The following demographic and clinical data have been recorded: sex, age, years of education, occupation (employed, unemployed, housewife/retired), body mass index (BMI), duration of psoriasis (years after diagnosis), presence of psoriatic arthritis and other comorbidities (hypertension, diabetes, psychiatric disorders), therapy for psoriasis. Each subject underwent the following questionnaires:

- Zung Self-rating Depression Scale (SDS): a 20-item self-administered questionnaire investigating the presence of depression-related affective, psychological, and somatic symptoms. Each item is scored from 1 to 4, and the subject must report how often they felt or behaved in a certain way (10 questions have a negative connotation and the others have a positive connotation), choosing from the following replies: “a little of the time”, “some of the time”, “good part of the time”, “most of the time”. The total score of the test is then converted into a *z* score. The questionnaire is reliable in terms of convergent validity and diagnostic discrimination [34,35]. A *z* score > 50 has been considered as a cut-off for depression.
- Pittsburgh Sleep Quality Index (PSQI): a 19-item self-report questionnaire assessing sleep quality (e.g., sleep latency, sleep disturbances, use of sleeping medication, daytime dysfunction) over a 1-month time interval. In addition, it is possible for the subject to report other conditions, not already mentioned in the PSQI, disturbing their sleep (open answer). Each item is scored on a 0–3 interval scale [36]. Different studies have set different cut-off scores for specific populations [37]. Since higher scores indicate poorer sleep quality, scores above the median value of the sample have been considered as a cut-off for poor sleep quality. The percentage of patients scoring ≥5 has been also reported, since this cut-off has been found to be useful in identifying poor sleepers in most studies [37].
- Interaction Anxiousness Scale (IAS): a 15-item self-rating scale assessing the level of distress when meeting and talking with other people. Some items have a positive connotation. Each item is scored on a five-point scale and the subject must answer to every statement choosing from the following replies: “not at all characteristic of me”, “slightly characteristic of me”, “moderately characteristic of me”, “very characteristic of me”, “extremely characteristic of me”. Higher scores relate to higher levels of interaction anxiety. The IAS has a good validity and internal consistency [38,39]. The scores above the median value of the sample have been considered as a cut-off for interaction anxiety.
- Audience Anxiousness Scale (AAS): a 12-item self-report survey assessing the level of audience anxiety in case of a public performance, from speaking in front of other people to “stage fright”. Some items have a positive connotation. Each item is scored on a five-point scale and the subject must answer to every statement choosing from the following replies: “not at all characteristic of me”, “slightly characteristic of me”, “moderately characteristic of me”, “very characteristic of me”, “extremely characteristic of me”. Higher scores relate to higher levels of audience anxiety. The AAS has a good validity and internal consistency [38]. Scores higher than the median value of the sample have been considered as a cut-off for audience anxiety.

In addition, each subject underwent a dermatological visit, and the severity of psoriasis was assessed by the Psoriasis Area Severity Index (PASI) [40].

When calculating the PASI score, the body is divided into four anatomical regions: Head (H), Upper Limbs (UL), Trunk (T), Lower Limbs (LL). For each anatomical region, the percentage of skin area involved and the severity of the disease are assessed and multiplied with one another to obtain a sub-score per anatomical region considered. The percentage of skin area involved (from 0% to 100%) is estimated and expressed in number (from 0 to 6) as follows:

0 = 0% of involved area;
1 = 1%–9% of involved area;
2 = 10%–29% of involved area;
3 = 30%–49% of involved area;
4 = 50%–69% of involved area;
5 = 70%–89% of involved area;
6 = 90%–100% of involved area.

The severity of the disease is assessed evaluating the following clinical signs: erythema, induration, and desquamation on a five-point scale with a score of 0 indicating none, 1 mild, 2 moderate, 3 severe, and 4 very severe. The proportion of the whole integument represented by each anatomical region is also considered. The sub-scores of the anatomical regions are then summed to obtain the PASI total score (ranging from 0 to 72). Higher scores indicate more severe conditions. The PASI is widely used in clinical practice and is a useful tool to assess the severity of psoriasis [41].

### 4.2. Statistical Analysis

Data were analyzed using the Statistical Package for Social Science (SPSS 17.0, SPSS Italia srl, Bologna, Italy). Quantitative variables were described using mean and standard deviation. The difference between means was evaluated by the *t*-test. Qualitative variables were described using percentage. The difference between proportions was evaluated by the χ-square test. Pearson’s correlation was performed to evaluate possible correlations between physical or dermatological-related variables (PASI total score, duration of psoriasis, BMI), psychiatric-related variables (SDS, IAS, AAS), and sleep quality (PSQI). Unconditional logistic regression was performed to evaluate possible associations between psychiatric variables (SDS, IAS, AAS), dermatological variables [(PASI total score but also clinical signs of psoriasis considered alone: erythema, induration, desquamation of the H, UL, T, LL), disease duration, psoriatic arthritis, therapy], sleep quality (PQSI), comorbidities, and demographic characteristics (sex, age, years of education, occupation, BMI).

## 5. Conclusions

In light of what has been reported here, it is apparent that psoriasis is linked to depression, interaction and audience anxiety, as well as to poor sleep quality. Hence, psoriatic patients should be assessed from a holistic point of view, in order to identify associated disorders that could benefit from targeted treatments. Other studies with a larger sample size and a healthy control group are necessary to confirm our results.

## Figures and Tables

**Table 1:** General characteristics of the patients (*n*: 102).

Variables	Values
Age	48.7 ± 13.6
Female	49, 48%
Male	53, 52%
Ethnicity: Caucasian	101, 99%
Ethnicity: Indian	1, 1%
BMI	27.3 ± 5.3
Years of education	10.6 ± 3.8
**Comorbidity**	
Hypertension	30, 29.4%
Diabetes	10, 9.8%
Anxiety	5, 4.9%
**Working characteristics**	
Unemployed	13, 12.7%
Employed	59, 57.8%
Housewife/retired	30, 29.4%

The table shows the general characteristics of the sample. The values are expressed as mean ± standard deviation (SD) or number and percentage (%). BMI: Body Mass Index.

**Table 2:** IAS and PASI.

PASI Score	OR	95% CI	*p*-Value
**Head**			
Erythema	1.9	0.74–4.99	0.175
Induration	0.8	0.34–2.05	0.705
Desquamation	0.7	0.30–1.68	0.441
**Upper limbs**			
Erythema	1.0	0.54–1.96	0.921
Induration	0.95	0.42–2.16	0.915
Desquamation	1.2	0.54–2.60	0.657
**Trunk**			
Erythema	1.3	0.52–3.47	0.536
Induration	0.3	0.09–1.15	0.08
Desquamation	2.4	0.81–7.10	0.11
**Lower limbs**			
Erythema	2.8	1.08–7.2	0.033
Induration	0.5	0.19–1.13	0.09
Desquamation	1.5	0.68–3.19	0.319

The Table shows the association between IAS and PASI scores. IAS: Interaction Anxiousness Scale; PASI: Psoriasis Area Severity Index.

**Table 3:** PSQI and PASI score.

PASI Score	OR	95% CI	*p*-Value
**Head**			
Erythema	14.8	1.4–147.2	0.021
Induration	0.6	0.23–1.47	0.258
Desquamation	1.1	0.46–2.67	0.805
**Upper limbs**			
Erythema	1.6	0.81–3.33	0.163
Induration	0.9	0.37–2.07	0.777
Desquamation	0.4	0.21–1.11	0.08
**Trunk**			
Erythema	2.0	0.73–5.42	0.173
Induration	0.4	0.13–1.54	0.204
Desquamation	1.2	0.44–3.46	0.672
**Lower limbs**			
Erythema	1.6	0.80–3.18	0.183
Induration	0.7	0.32–1.71	0.498
Desquamation	0.9	0.42–1.87	0.758

The table shows the association between PSQI and PASI scores. PSQI: Pittsburgh Sleep Quality Index; PASI: Psoriasis Area Severity Index.
